# Diagnostic Challenges of a Mass in the Left Atrium

**DOI:** 10.7759/cureus.8620

**Published:** 2020-06-14

**Authors:** Khaing Khaing Htwe, Thein T Aung

**Affiliations:** 1 Internal Medicine, Kingsbrook Jewish Medical Center, Brooklyn, USA; 2 Cardiology, Miami Valley Hospital, Wright State University, Dayton, USA

**Keywords:** intracardiac mass, left atrium, cardiac metastasis

## Abstract

A 61-year-old female former smoker with history of bronchial-associated lymphoid tissue lymphoma presented with increasing dyspnea, cough with white phlegm and significant weight loss. Chest X-ray showed complete opacification of the left hemithorax. A computed tomography (CT) pulmonary angiogram ruled out pulmonary embolism but revealed mass within the left atrium. A transthoracic echocardiography showed an echogenic mass in the left atrium. A cardiac MRI confirmed a bulky left lung mass suggestive of carcinoma invading the left atrium via the left pulmonary veins. CT-guided biopsy of left lung mass was suggestive of non-small cell lung carcinoma (NSCLC, adenocarcinoma). We would like to discuss the challenges and the importance of making the correct diagnosis of intracardiac mass.

## Introduction

A mass in the left atrium can be challenging to diagnose. The leading differential diagnoses include thrombus, vegetation and tumor, which may be primary (benign or malignant) or metastatic [1]. We would like to present a case of mass in the left atrium found in a 61-year-old woman which turned out to be metastatic invasion to the heart from the lesions in the lungs. Incidence of metastatic tumors of the heart is more than that of primary tumors. Careful consideration of differential diagnoses with the help of detailed clinical features and imaging studies is important. This report highlights the work-up leading to the diagnosis and the possible routes of spread to the heart.

## Case presentation

A 61-year-old female former smoker presented with increasing dyspnea and productive cough with white expectoration for a few days. She denied fever, chest pain, palpitation, orthopnea, paroxysmal nocturnal dyspnea and edema. She had unintentional weight loss of 20 lbs along with loss of appetite. She had past medical history of bronchial-associated lymphoid tissue (BALT) lymphoma and was treated with rituximab. A positron emission tomography (PET) scan two months prior to the admission showed left lower lobe mass with obstruction of the left mainstem bronchus, and diffuse bilateral pulmonary nodules with moderate left pleural effusion. Subsequently, she received chemotherapy with obintuzumab and bendamustine.

On admission, she was afebrile, sinus tachycardia 110/min, normotensive, respiratory rate 25/min and oxygen saturation 96% on 2 L nasal cannula oxygen. There was neither jugular venous distension nor pedal edema. Cardiac auscultation revealed normal S1 and S2 without murmur or added sound. Lung auscultation revealed diminished breath sound over the left hemithorax. The rest of the physical examination was unremarkable.

**Figure 1:**
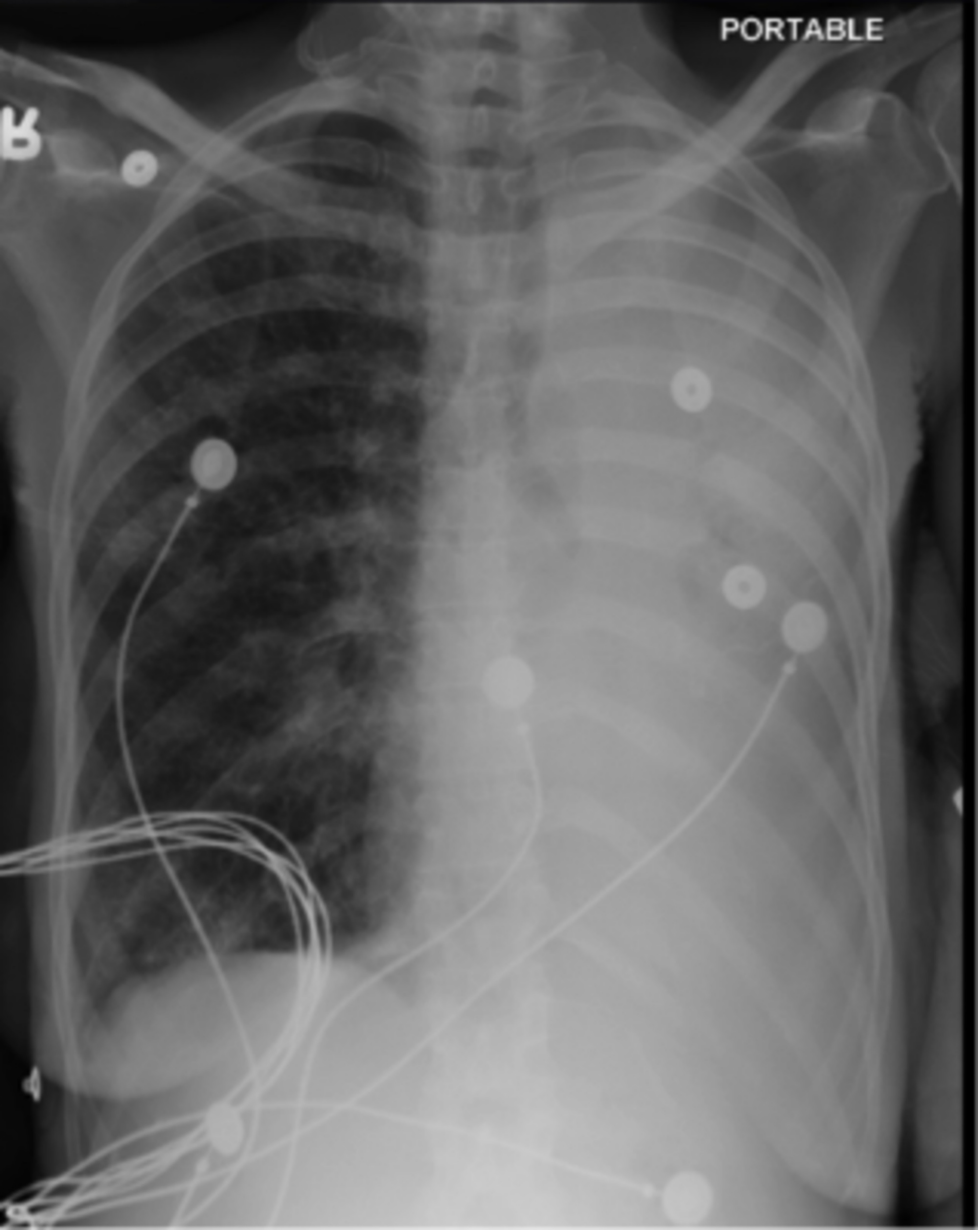
Chest X-ray showing opacification of the left hemithorax

Her electrocardiogram showed sinus tachycardia with the rate of 109/min. Complete blood count showed normocytic anemia with hemoglobin 9 g/dl which was stable compared to that two months ago. Blood chemistry was within normal limits. Chest X- ray showed opacification of the left hemithorax (Figure 1).

**Figure 2:**
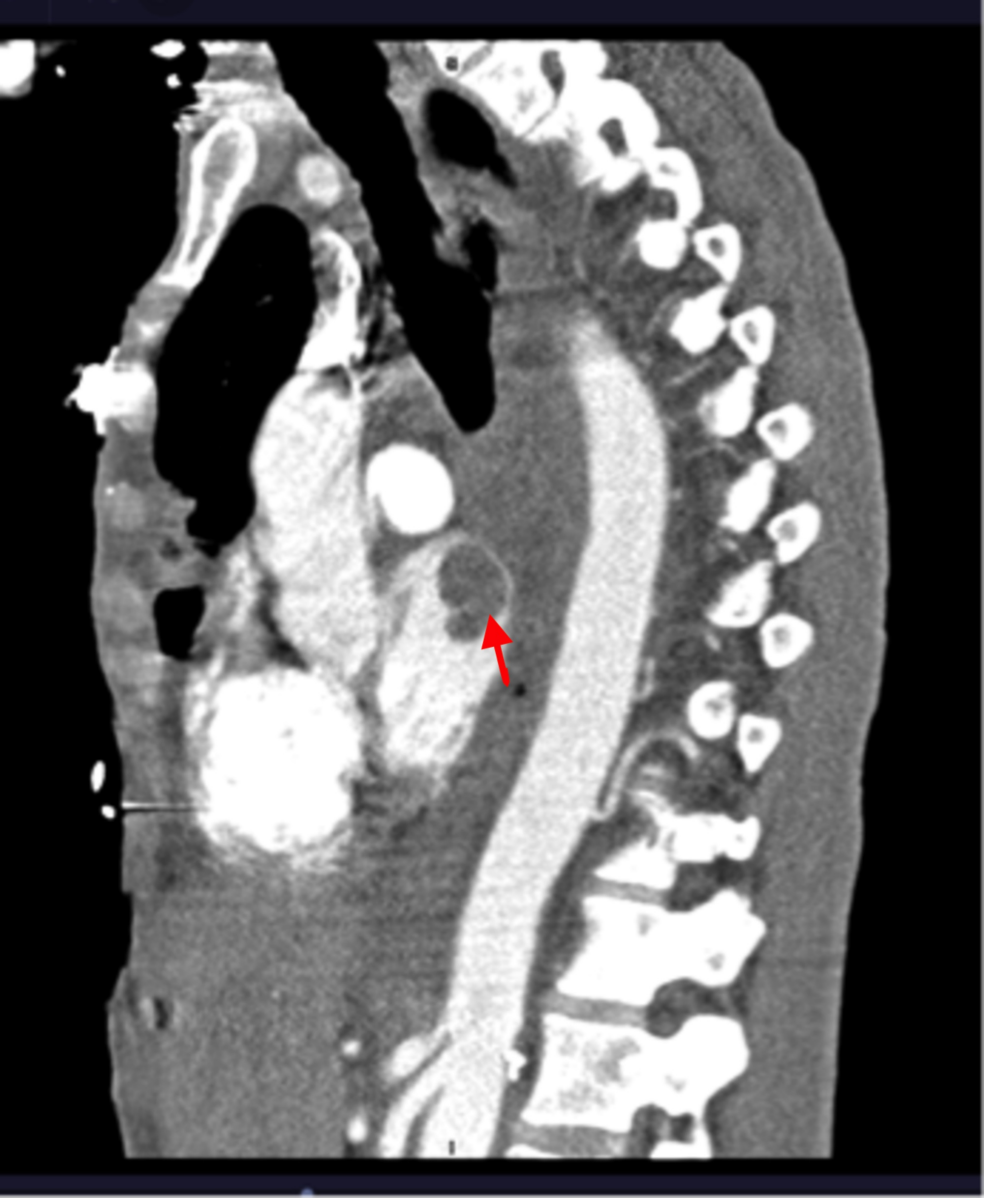
CT angiography of the chest showing tumor invasion to the left atrium

Given these observed findings along with the elevated risk of thrombosis in active cancer, computed tomography (CT) pulmonary angiogram was performed (Figure 2). CT chest ruled out pulmonary embolism but revealed a large filling defect within the left atrium, left inferior and superior pulmonary veins. It was suspected to be a thrombus since it was not reported on earlier PET scan. Apixaban was initiated given the concern for thromboembolism.

**Figure 3:**
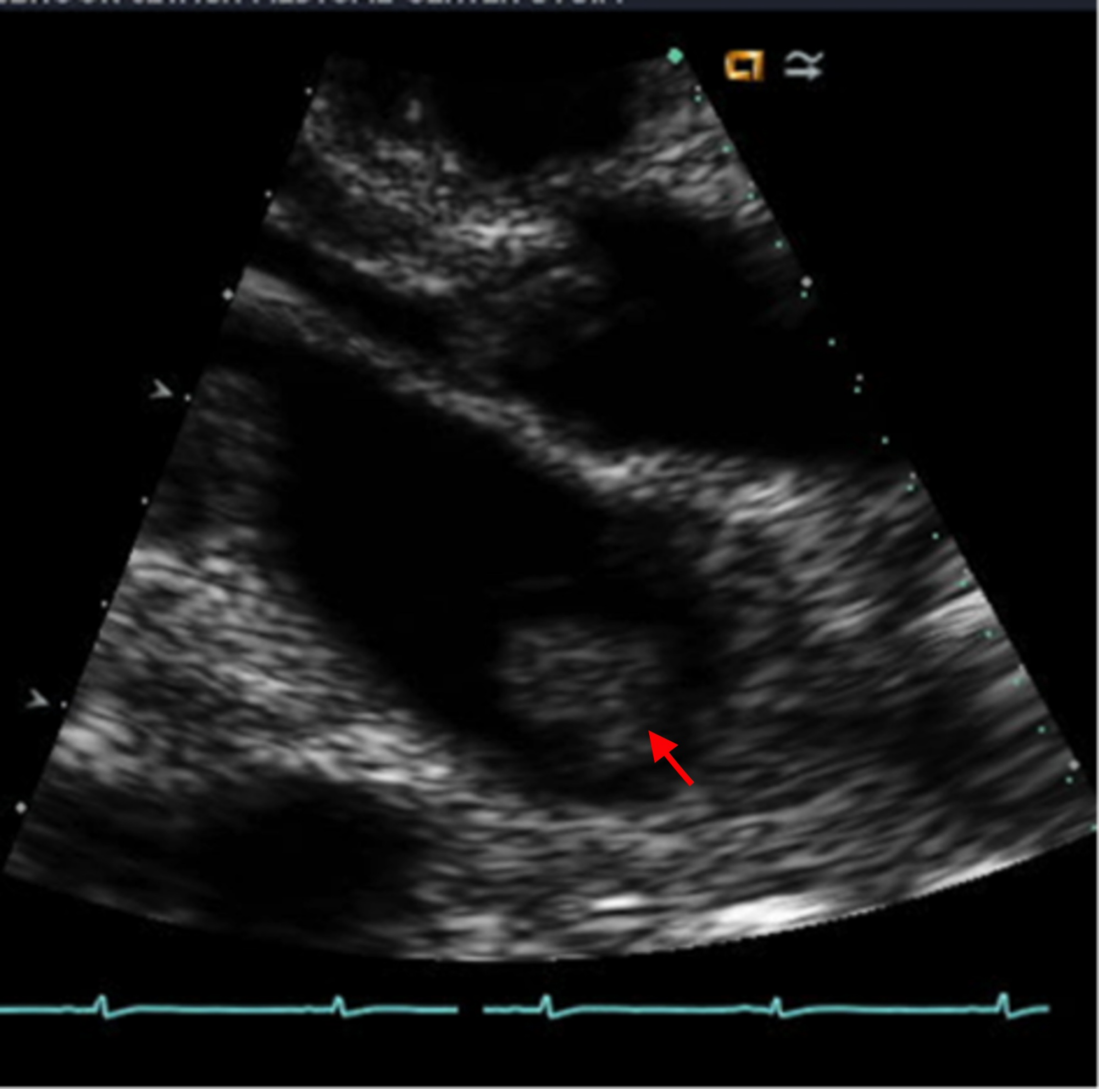
Transthoracic echocardiogram showing the left atrium mass

A transthoracic echocardiogram showed a 3 x 3 cm, mobile, echogenic mass in the left atrium best seen in the parasternal long axis view (Figure 3). Her left atrial size was reported to be normal. Her left ventricular size and function were also within normal limit. Further evaluation with transesophageal echocardiogram (TEE) was considered. However, the patient did not provide consent for TEE after being informed about the risks of the procedure. 

**Figure 4:**
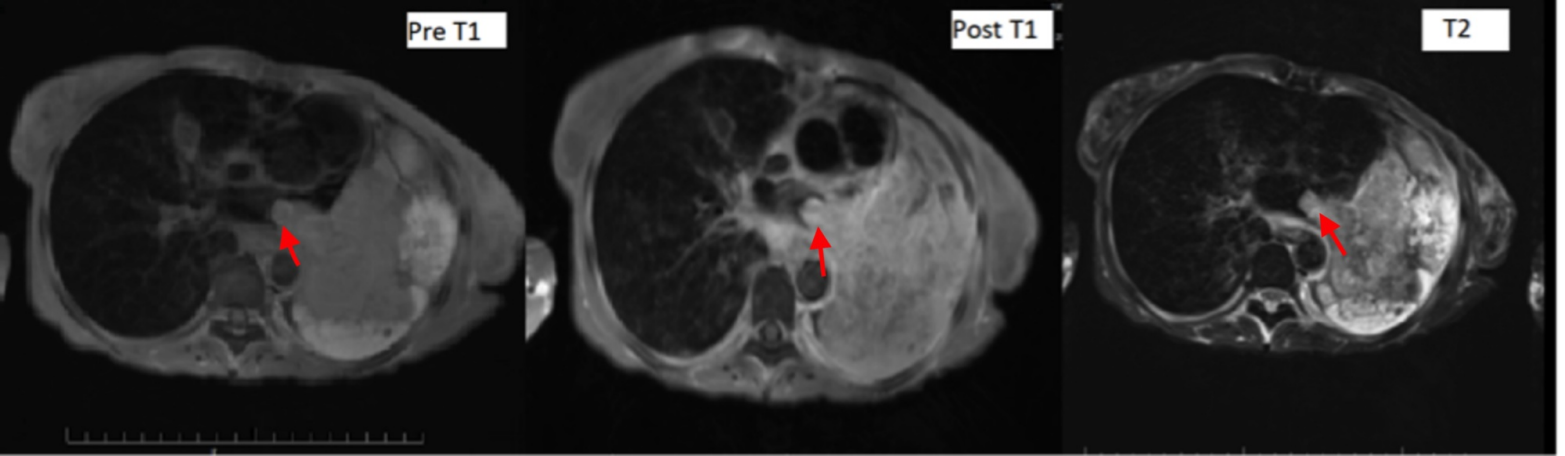
MRI of the heart showing tumor invasion to the left atrium

To offer the best characterization of soft tissue cardiac masses, cardiac magnetic resonance imaging (CMR) was performed (Figure 4). It confirmed a bulky left lung mass suggestive of carcinoma invading the left atrium via the left pulmonary veins. There was bulky intraluminal tumor thrombus and extensive mediastinal invasion by tumor. CT-guided biopsy of left lung mass was suggestive of non-small cell lung carcinoma (NSCLC). A diagnosis of dual synchronous cancers, BALT lymphoma and NSCLC, adenocarcinoma to be exact, with cardiac invasion was made. As of now, we are not sure about the relationship between these two tumors. We do not know which one had invaded the left atrium. She continued to receive palliative chemotherapy. After discussion with the hemato-oncology, the anticoagulation was stopped due to the risk of internal bleeding. 

## Discussion

Pericardium is avascular fibrous membrane that provides resistance to direct tumor extension. Endocardium and myocardium layers have low rate of cell division. Because of those two reasons, we tend to think that cardiac tissue is relatively resistant to tumor metastasis and metastatic cardiac tumor is rare in clinical practice. However, the incidence reported in literature is higher than expected. Cardiac metastasis was seen in up to 21% of postmortem patients who had died of malignancies [2]. Malignant melanomas are particularly notorious for cardiac metastasis by bloodstream seeding. This is also found in leukemia and lymphoma. Another way is direct extension. Solid cancers from nearby organs, such as lung, breast, soft tissue sarcoma, renal carcinoma and hepatocellular carcinoma, usually invade cardiac tissue.

Our patient had dual synchronous carcinomas: BALT lymphoma and NSCLC, adenocarcinoma. BALT lymphoma is a low-grade primary B-cell lymphoma, classified as an extranodal marginal zone B-cell lymphoma of mucosa-associated lymphoid tissue (MALT) type. It is the most common type of primary pulmonary lymphoma. However, it is a rare entity accounting for less than 1% of all lymphomas. BALT lymphoma is associated with Sjogren’s syndrome, rheumatoid arthritis, systemic lupus erythematosus and infections.

Lung adenocarcinoma usually arises from peripheral small bronchi or alveolar epithelial cells. The incidence of adenocarcinoma of lung has risen dramatically. Synchronous primary malignancy and extranodal marginal zone lymphoma in the same anatomical sites are very unusual [3]. Concurrent NSCLC and BALT lymphoma have been scarcely reported in the literature. There is no identified cause favoring the simultaneous occurrence of these different tumors. There are speculations that a genetic linkage between the API2-MALT1 fusion gene and the high incidence of trisomy 3 seen in MALT lymphoma may coexist with the development of lung adenocarcinoma [4]. One case report speculated that cigarette smoke leads to BALT hyperplasia, which is then followed by neoplastic transformation [5]. Since our patient was a former heavy smoker, the fact that smoking being the possible cause of these synchronous tumors can be intensified.

Lung cancers may spread to cardiac tissue through retrograde lymphatic spread, hematogenous dissemination, transvenous extension and direct extension. The pericardium is, obviously, the most commonly involved cardiac structure, up to 69% of all cardiac metastasis. Epicardial (25%-34%) and myocardial involvement (29%-32%) represent the second and third most common sites, respectively [6]. Endocardial involvement is very rare, likely due to the low rate of cell division in cardiac tissue. Our patient had the tumor invasion to the left atrium through the pulmonary veins, which is an extremely rare event.

The clinical manifestations of metastatic cardiac tumor can be vague and non-specific. It usually depends on the location of cardiac metastasis and underlying tumor burden. It may remain asymptomatic until being detected on autopsies. Medical emergencies, such as cardiac tamponade, heart failure, myocardial infarction and brady- or tachyarrhythmia, can occur [2]. 

Our patient presented with vague symptoms: dyspnea, cough and weight loss. Electrocardiogram (EKG) revealed sinus tachycardia. CT angiogram picked up the intracardiac mass. Echocardiogram is generally preferred for initial evaluation of a cardiac mass as it is an accessible, non-invasive and low-cost imaging modality. The diagnosis of metastatic cardiac tumor is challenging. Intracardiac masses are frequently considered to be thrombi. It is also difficult to differentiate thrombi from metastatic tissue solely based on imaging studies. If the mass is relatively homogenous and the patient has hypercoagulability, then the diagnosis is more likely to be thrombus. If the mass is heterogeneous with nearby tumor growth, it tends out to be suggestive of carcinoma. However, in real world, malignancy is thrombogenic and these two diagnoses are not mutually exclusive. Cardiac CT and CMR provide more detailed information. CMR can better characterize the morphologic features, architecture and perfusion of a cardiac mass. CMR with contrast enhancement can help to distinguish a tumor from a thrombus [7]. Tumors with the epicardial involvement require coronary angiography preoperatively to define potential involvement of the coronary arteries which could be fatal from myocardial ischemia, and also determine the coronary blood supply of the tumor. In our case, if we had not made the correct diagnosis and focused on intracardiac thrombus, she might have ended up with empiric anticoagulation without further evaluation. That could lead to devastating results, such as bleeding or even pericardial tamponade. We discussed with the hemato-oncology and then made an informed decision to stop the anticoagulation.

## Conclusions

The clinical manifestations of cardiac metastasis are relatively uncommon, but the implications are critical. It typically occurs in patients who have multiple metastasis and high disease burden. Clinicians should always be aware of the possibility of cardiac metastasis in patients with known malignancy or a suspicious cardiac mass found incidentally on imaging studies. Meticulous workup and accurate diagnosis are required to avoid potentially life-threatening complications such as pericardial tamponade.
